# In Situ Electrochemical Monitoring of the Crevice Corrosion Process of the 7075-T651 Aluminium Alloy in Acidic NaCl and NaNO_3_ Solution

**DOI:** 10.3390/ma16072812

**Published:** 2023-03-31

**Authors:** Shengjie Wang, Yamin Cao, Xiaohang Liu, Guangyi Cai

**Affiliations:** 1 National Key Laboratory of Science and Technology on Electromagnetic Energy, Naval University of Engineering, Wuhan 430033, China; 2 East Lake Laboratory, Wuhan 420202, China; 3 Marine Equipment Inspection & Testing Co., Ltd., Qingdao 266200, China; 4 Goertek Optical Technology Co., Ltd., Weifang 261041, China

**Keywords:** crevice corrosion, pH sensors, electrochemical monitoring, high strength aluminium alloy

## Abstract

The crevice corrosion of the 7075-T651 aluminium alloy was investigated using in situ electrochemical impedance spectroscopy (EIS), potentiodynamic polarization curves (PC), and H^+^ sensors in acidic NaCl solution with different contents of NaNO_3_. In the solution without NaNO_3_, the pH in the crevice increased rapidly and gradually reached a relatively stable status. The corrosion of the aluminium alloy in the crevice was inhibited and crevice corrosion could not be initiated. In the solution with NaNO_3_, the pH increased rapidly at the initial immersion period and then decreased gradually. The corrosion of the aluminium alloy inside the crevice could be enhanced and the corrosion of the aluminium alloy outside crevice could be inhibited. This triggered crevice corrosion in the solution with NaNO_3_. The inhibited corrosion outside the crevice can be attributed to the improved passive film of the specimen outside the crevice by nitrate. The accumulated secondary products of ammonia inside the crevice led to selective dissolution of copper, which triggered the nucleation of pitting corrosion and promoted the corrosion of the specimen inside the crevice.

## 1. Introduction

High-strength aluminium alloys, with high strength and low density, are among the most important materials for conveyance to achieve lightweight, and it has been widely applied in the transportation industry. However, high-strength aluminium alloys are vulnerable to localized attack in corrosion media containing chloride ions [1,2,3,4]. An aluminium alloy is more susceptible to localized corrosion (namely crevice corrosion) when its surface is covered with crevice structures such as rivets, lap joints, and thread connections [5,6,7]. Crevice corrosion is initiated due to the change in the composition of the solution inside the crevice. It has been reported that a concentration difference in metal ions [8], dissolved oxygen [9], H^+^, and Cl^−^ [10,11] could be formed. This leads to the macro separation of anode and cathode, and then drives the active dissolution of metal inside crevice. Once crevice corrosion is initiated, serious localized defects could be formed in a relatively short period. The strength of materials decreases drastically after the occurrence of crevice corrosion. This poses a significant threat to the service life of high-strength aluminium alloy components.

Various attempts have been made to inhibit the corrosion of aluminium and its alloys [12,13,14,15,16]. It has been reported that NO_3_^−^ can be considered an effective inhibitor for aluminium and its alloys. Li et al. [15] reported that NaNO_3_ promoted the formation of thick oxide films on AA1085 and then re-passivated the pitting corrosion. Gao et al. [17] found that NaNO_3_ significantly improved the properties of an oxide film, and the localized attack was completely inhibited when the concentration of NaNO_3_ was above 0.03 M. However, the role of NO_3_^−^ on the corrosion of aluminium alloys is still controversial. Balaskas et al. [18] observed that a low concentration of NO_3_^−^ inhibited the corrosion of the 2024 aluminium alloy, but was susceptible to localized attack at a high concentration of NO_3_^−^. The deterioration of the inhibiting properties at high concentrations was attributed to the presence of the NO_3_^−^ ions that promoted the redeposition of copper at the cathodic sites. Blanc et al. [19] proposed that NH_3_ could selectively dissolve copper and induce pitting nucleation due to the reduction of NO_3_^−^. The concentration of NO_3_^−^ inside the crevice decreased gradually due to the reduction reaction and the limited diffusion process when the crevice was formed on the aluminium alloy surface. This improved the performance of a passive film inside the crevice and outside the crevice. However, the secondary product NH_3_ progressively accumulated in the crevice solution, which affected the corrosion of the aluminium alloy inside the crevice. This indicated that the effect of NO_3_^−^ on the corrosion of the aluminium alloy inside the crevice was different from that outside the crevice in a corrosive medium. Thus, it is essential to ascertain the effect of NO_3_^−^ on crevice corrosion of high-strength aluminium alloys.

In this study, the crevice corrosion behaviour of the 7075-T651 aluminium alloy in an acidic NaCl solution with various concentrations of NaNO_3_ was systematically studied. The corrosion behaviour and comparison of the aluminium alloy inside and outside the crevice was analysed using electrochemical measurements and surface analysis. In addition, the underlying crevice corrosion mechanism in the solution with NO_3_^−^ is discussed.

## 2. Materials and Methods

### 2.1. Materials and Solutions

The used material was 7075-T651 (Simon Metal Materials Co., Ltd., Shanghai, China) and its composition was as follows: Zn 5.63 wt.%, Mg 2.45 wt.%, Cu 1.55 wt.%, Si 0.045 wt.%, Fe 0.18 wt.%, and Al balance. The sizes of the working electrodes used were 10 × 10 × 5 mm^3^ and 20 × 10 × 5 mm^3^. The electrode with 10 × 10 × 5 mm^3^ was applied for electrochemical measurements and the 20 × 10 × 5 mm^3^ electrode for morphology analysis. Prior to tests, the working electrodes were polished with 1500 grit silicon carbide paper, then washed with deionized water, and degreased with acetone (Sinopharm Chemical Reagent Co., Ltd., Shanghai, China). The test solution was a 3.5 wt.% NaCl solution (pH = 2), without and with different concentrations of NaNO_3_ (0 wt.%, 0.2 wt.%, and 0.5 wt.%). All tests were performed at a temperature of 30 °C.

### 2.2. Configuration of Crevice and Experimental Device

**Figure 1:**
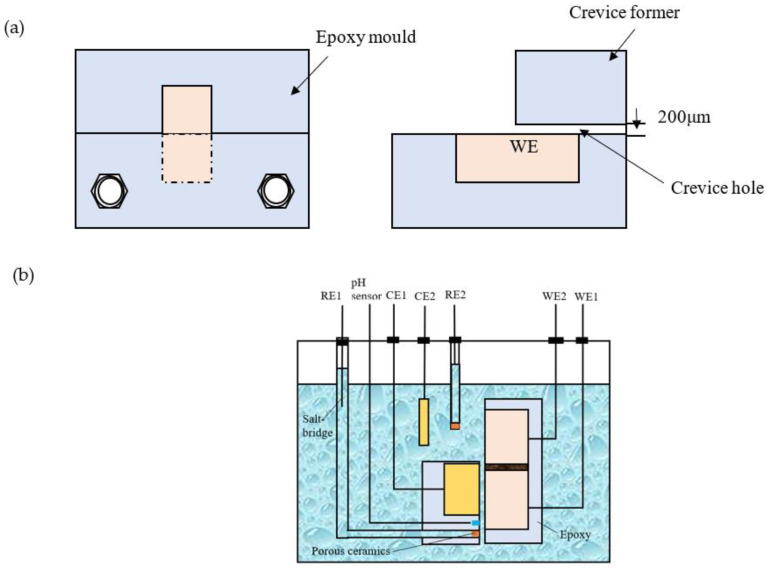
Configuration of the crevice and sketch map of the setup. (**a**) Crevice configuration for morphology analysis; (**b**) crevice configuration and sketch map of the setup for electrochemical tests.

Figure 1 shows the crevice structure and test setup. The crevice configuration that is shown in Figure 1a, consisting of one electrode with an exposed area of 2 cm^2^, was used for morphology analysis. One half of the electrode was inside the crevice, and another half was outside the crevice. The crevice structure and test setup used for the electrochemical test, shown in Figure 1b, consisted of two identical 7075-T651 electrodes with an exposed area of 1 cm^2^. One electrode (WE1) was placed inside the crevice, and another one (WE2) was located outside the crevice. A Pt/IrOx electrode was embedded in the crevice former and was used to measure the concentration of H^+^ of inside the crevice. The inner counter electrode (CE1) and reference electrode (RE1) were used for the electrochemical measurements of WE1, while the outer counter electrode (CE2) and reference electrode (RE2) were used for the electrochemical measurements of WE2. A PTFE gasket with 200 μm thickness is installed between two epoxy modules to create a 200 μm crevice.

According to the protocol in reference [20], the Pt/IrOx electrodes were prepared with the following steps. First, 50 mL 1.5 g/L IrCl_4_·xH_2_O was prepared and 0.5 mL of aqueous 30% H_2_O_2_ was added. Then, the solution was stirred for 10 min. Next, 0.25 g of oxalic acid (C_2_H_2_O_4_) was added and the solution was stirred for another 10 min. Gradually anhydrous potassium carbonate was added to the solution until the pH was slowly raised to 10.5. The electrodeposition process was -conducted using cyclic voltammetry (CV) scanning between 0.2 and 0.75 V (vs. Ag/AgCl) with a scanning rate of 50 mV/s for 100 cycles and then heat treatment was carried out at 100 °C for 2 h.

**Figure 2:**
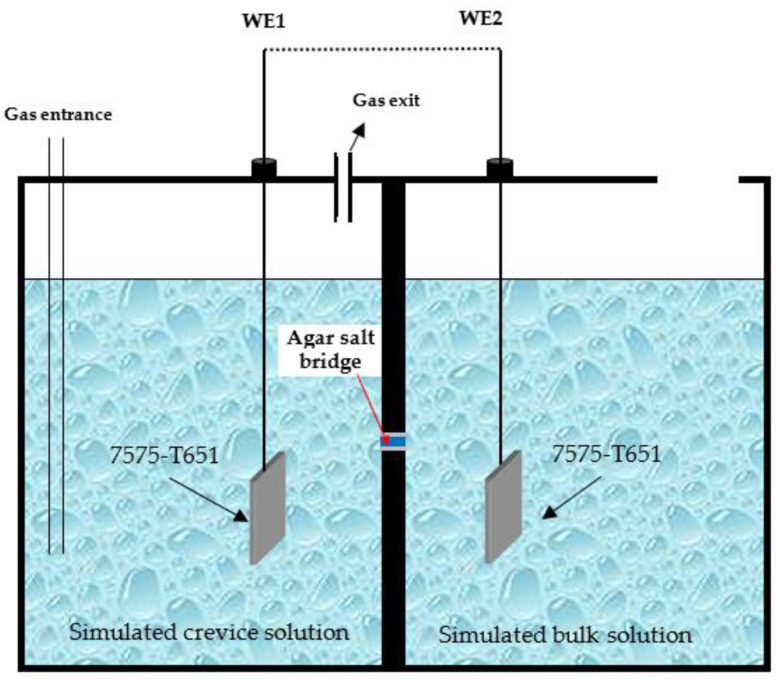
Schematic diagram of the two-chamber setup for corrosion morphology analysis in simulated crevice solution and bulk solution.

The schematic diagram of the setup shown in Figure 2 was used to figure out the effect of chemical species on crevice corrosion of the 7075-T651 aluminium alloy. This setup consisted of two chambers, and each chamber contained a 7075-T651 specimen. One of the chambers was used to simulate the crevice environment, and another was used to simulate the bulk solution. These two chambers were connected by a salt bridge. The simulated bulk solution was exposed to air. However, the simulated crevice solution was purified using N_2_ during the test, because the oxygen inside the crevice should be depleted due to the limited mass transfer processes by the narrow crevice opening size. These two specimens were always connected in the test.

### 2.3. Electrochemical Tests

A CS 330 electrochemical workstation (CorrTest, Wuhan, China) was used to explore the electrochemical corrosion behaviour of the 7075-T651 aluminium alloy. Electrochemical impedance spectroscopy (EIS) was measured over a frequency range from 100 kHz to 10 mHz using a 5 mV amplitude sinusoidal voltage. The electrochemical measurements were conducted using an electrochemical workstation. The cathodic reactions were hardly inhibited. Therefore, the potentiodynamic polarization curve of the specimen inside the crevice was recorded at a scan rate of 0.5 mV/s starting from −25 mV against open circuit potential (OCP). Correspondingly, the specimen outside the crevice was measured starting from −150 mV to 150 mV against OCP.

### 2.4. Morphology Analysis

A digital camera was used to acquire optical images of 7075-T651 after crevice corrosion. The surface microstructure and cross-sections of the crevice specimen were characterized using scanning electron microscopy (SEM). The elemental composition of the localized corrosion area inside the crevice was analysed using energy dispersive spectroscopy (EDS). The 3D morphology of the specimens after removing the corrosion products was detected using a 3D ultra-depth microscope.

## 3. Results

### 3.1. OCP Tests

**Figure 3:**
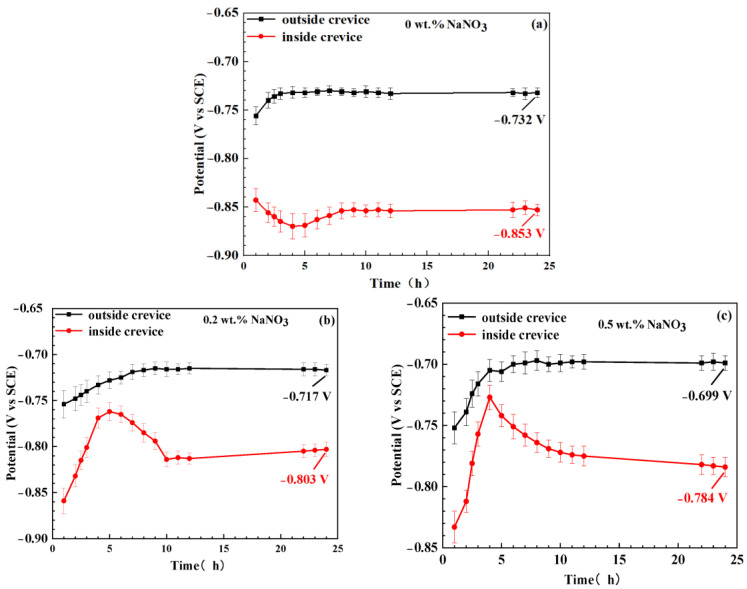
The OCPs of specimens inside and outside the crevice in solutions with different concentrations of NaNO_3_ after corrosion for 24 h. (**a**) 0 wt.% NaNO_3_, (**b**) 0.2 wt.% NaNO_3_, (**c**) 0.5 wt.% NaNO_3_.

Figure 3 exhibits the OCP of the specimen inside and outside the crevice in solution with various contents of NaNO_3_. It can be seen that the OCPs outside the crevice are always more positive than those of specimens inside crevice. This is mainly attributed to the inhibited cathodic reactions inside crevice. The OCPs of the specimens outside the crevice initially shift in the positive direction and reach relatively stable values in the solutions with various concentrations of NaNO_3_. Then, a significant potential difference between the alloy inside and outside the crevice is formed in the different test solutions. The alloy inside the crevice acts as anode and the alloy outside crevice works as cathode. However, the OCPs of the specimens inside the crevice show different evolution tendencies in the solutions with different concentrations of NaNO_3_. In the solution without NaNO_3_, the OCPs move to the negative direction within 4 h of immersion; then shift to the positive direction and reach relatively stable values after immersion for 8 h. However, in the solutions with NaNO_3_, the OCPs move to the positive direction within 4 h of immersion, then shifts to the negative direction, and reach relatively stable values after immersion for 11 h. It has been acknowledged that the corrosion potential is determined by cathodic and anodic reaction kinetic processes [21,22,23]. The cathodic reactions inside the crevice are inhibited due to the depleted cathodic reactants. Thus, the evolution of OCPs of the specimen inside the crevice could represent the anodic reaction kinetic behaviour of the specimen inside the crevice. The enhanced anodic reaction results in the negative shift of OCPs; on the contrary, the OCPs move in the positive direction. This indicates that the dissolution of the aluminium alloy inside the crevice increases during the initial 4 h of immersion and is gradually inhibited after 4 h in the solution without NaNO_3_. The OCPs of the specimen inside the crevice rapidly shift to the positive direction during 4 h of immersion in the solution with NaNO_3_, indicating an inhibited anodic reaction rate. This can be attributed to the promotion of passive film growth on the specimen surface by NO_3_^−^ inside the crevice in a solution containing NaNO_3_. The negative displacement of OCPs suggests an enhanced dissolution of the specimen inside the crevice after 4 h of immersion, which can be related to the degradation of the passive film inside the crevice.

### 3.2. Potentiodynamic Polarization Curve Tests

**Figure 4:**
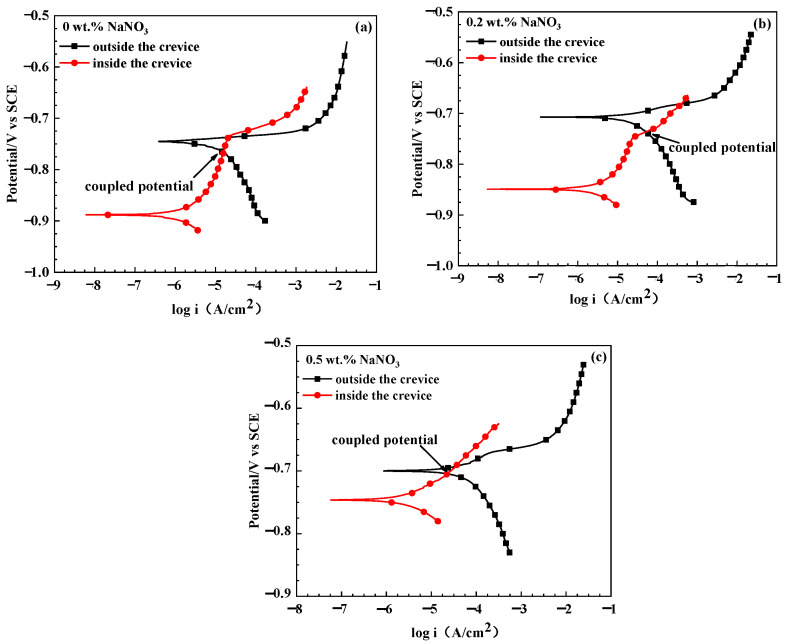
The polarization curves of specimens inside and outside the crevice in solutions with different concentrations of NaNO_3_ after corrosion for 24 h. (**a**) 0 wt.% NaNO_3_, (**b**) 0.2 wt.% NaNO_3_, (**c**) 0.5 wt.% NaNO_3_.

Figure 4 shows the polarization curves of WE1 and WE2 in the solutions with different concentrations of NaNO_3_ after 24 h of immersion. In the solution without NaNO_3_, the polarization curve of WE1 shows passive–active transition characteristics. This implies the passive film of WE1 is stable at corrosion potentials ranging from −0.895 V to −0.738 V. However, WE2 shows active corrosion characteristics, indicating that the passive film on the surface of WE2 was broken after 24 h of immersion. Furthermore, it can be seen that the coupled potential (the potential at the intersection of the anodic polarization curve of WE1 and the cathodic polarization curve of WE2) lies in the passive region (−0.895 V~−0.738 V) of the polarization curve of WE1. This indicates that the passive film of WE1 is stable when the specimens inside and outside the crevice are coupled together. Thus, 7075-T651 is not susceptible to crevice corrosion in the solution without NaNO_3_ after 24 h of immersion. In the solution with 0.2 wt.% NaNO_3_, it can be seen from Figure 4b that the passive film of WE1 is stable at a potential range from −0.848 V to −0.748 V. The stable potential range of the passive film is narrower in the solution with 0.2 wt.% NaNO_3_ than in the solution without NaNO_3_. Furthermore, the current density (at −0.82 V) of WE1 is about 28 μA in the solution without NaNO_3_ and 77 μA in the solution with 0.2 wt.% NaNO_3_. This indicates the performance of the passive film inside the crevice becomes poorer in the solution with 0.2 wt.% NaNO_3_ than it is in the solution without NaNO_3_ after 24 h of immersion. Moreover, it can be seen from Figure 4b that the coupled potential exceeds the pitting corrosion breakdown potential of the passive film of WE1. Thus, the passive film inside the crevice can be broken when WE1 and WE2 are connected. This means that 7075-T651 is susceptible to crevice corrosion in the solution with 0.2 wt.% NaNO_3_ after 24 h of immersion. In the solution with 0.5 wt.% NaNO_3_, the anodic current density of WE1 increases rapidly with increased anodic polarized potential. This indicates that the passive film on the specimen inside the crevice was broken, initiating its susceptibility to crevice corrosion in the solution with 0.5 wt.% NaNO_3_ after 24 h of immersion. Thus, it can be concluded from Figure 4a–c that NaNO_3_ significantly promotes the corrosion of 7075-T651 inside the crevice.

### 3.3. EIS Tests

**Figure 5:**
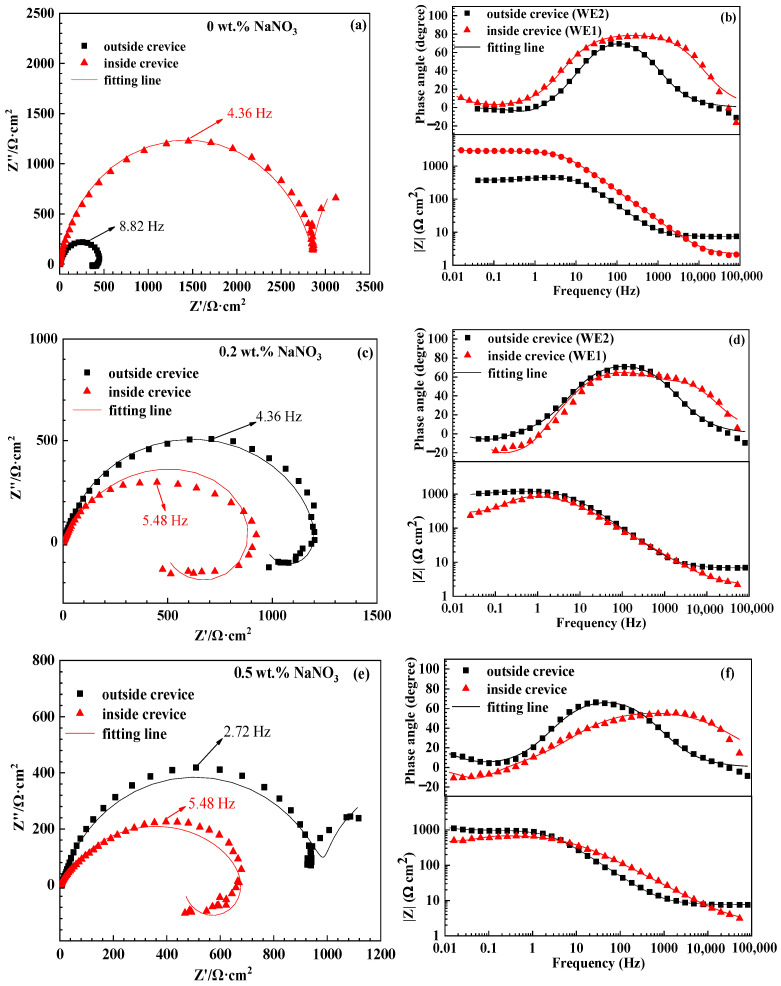
The EIS of specimens inside and outside the crevice in solutions with different concentrations of NaNO_3_ after corrosion for 24 h. (**a**,**b**) 0 wt.% NaNO_3_, (**c**,**d**) 0.2 wt.% NaNO_3_, (**e**,**f**) 0.5 wt.% NaNO_3_.

**Figure 6:**
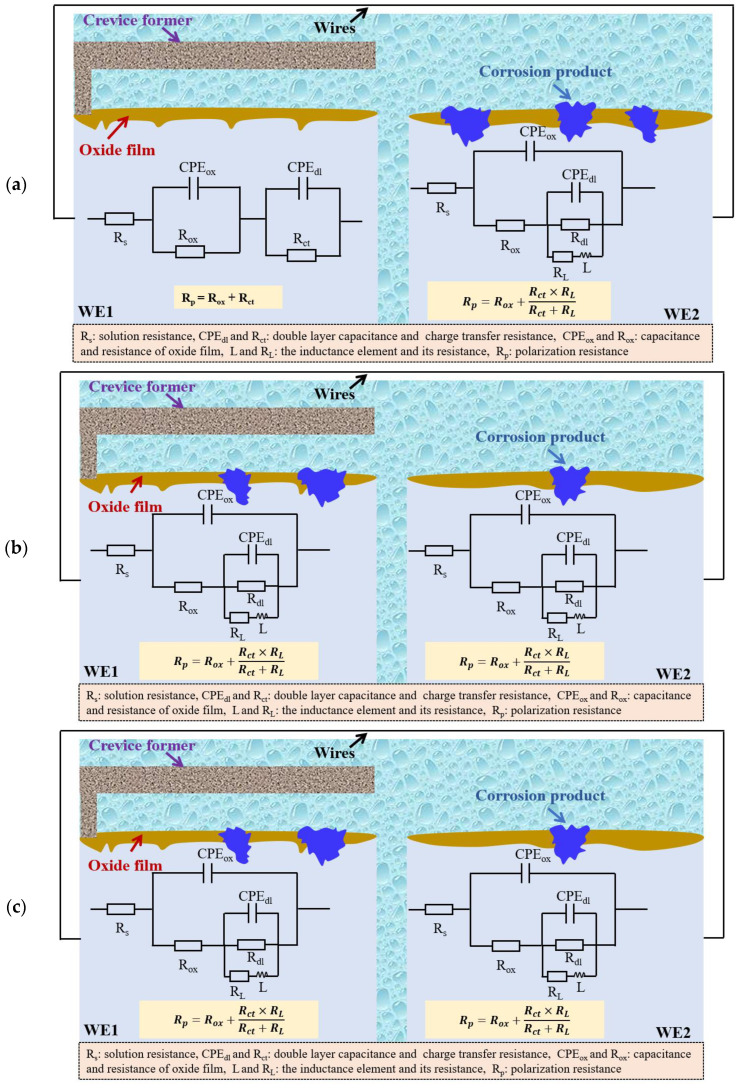
The interfacial structures and equivalent circuits of WE1 and WE2 specimens in solutions with different concentrations of NaNO_3_ after corrosion for 24 h. (**a**) 0 wt.% NaNO_3_, (**b**) 0.2 wt.% NaNO_3_, (**c**) 0.5 wt.% NaNO_3_.

The EIS plots of WE1 and WE2 in the solutions with different concentrations of NaNO_3_ after 24 h of immersion are shown in Figure 5. The corresponding equivalent circuits and interfacial structures between substrate and solution are shown in Figure 6. It can be observed in Figure 5a, that the impedance spectrum of WE1 contains two capacitance loops in the solution without NaNO_3_, where the capacitance loop at high frequencies can be ascribed to the oxide film. In contrast, the capacitance loop at low frequencies can be attributed to the double layer. Then, the interfacial structure of WE1/solution and the equivalent circuit are shown in Figure 6a. The impedance spectrum of WE2 is characterized by two capacitance loops and an inductance loop at low frequencies in the solution without NaNO_3_, as shown in Figure 5a. The inductance loop at low frequencies is related to localized corrosion of the aluminium alloy [24,25]. The two capacitance loops result from the oxide film and the charge transfer process. Then, the interfacial structure of WE2 in solution and the equivalent circuit are shown in Figure 6a in the solution without NaNO_3_. The impedance spectra of WE1 and WE2 contain two capacitance loops and an inductance loop in the solution with 0.2 wt.% NaNO_3_, which is similar to that of WE2 in the solution without NaNO_3_. This indicates that localized corrosion can be initiated on both WE1 and WE2. However, the diameter of the capacitance of WE1 is smaller than that of WE2 in the solution with 0.2 wt.% NaNO_3_, suggesting the occurrence of crevice corrosion. The interfacial structure of WE1(WE2) in solution and the equivalent circuits are shown in Figure 6b. The impedance spectrum of WE1 contains two capacitance loops and an inductance loop in the solution with 0.5 wt.% NaNO_3_. This indicates the occurrence of localized corrosion of the specimen inside the crevice. The impedance spectrum of WE2 consists of two capacitance loops similar to that of WE1 in the solution without NaNO_3_, indicating an oxide film covers on the specimen surface. The interfacial structures of WE1(WE2) in solution and equivalent circuits are shown in Figure 6c. The fitted results are listed in Table 1. It can be observed that the Rp of WE2 is smaller than that of WE1 in the solution without NaNO_3_. However, the Rp of WE2 is larger than that of WE1 in the solution with NaNO_3_. This demonstrates that crevice corrosion can be initiated in the solution with NaNO_3_ and could not occur without NaNO_3_ after 24 h of immersion. Furthermore, the Rp of WE1 decreases, and the Rp of WE2 increases with an increased concentration of NaNO_3_. This indicates increased corrosion of the specimen inside the crevice and inhibited corrosion of the specimen outside the crevice as the concentration of NaNO_3_ increases.

### 3.4. pH Concentration Measurement

**Figure 7:**
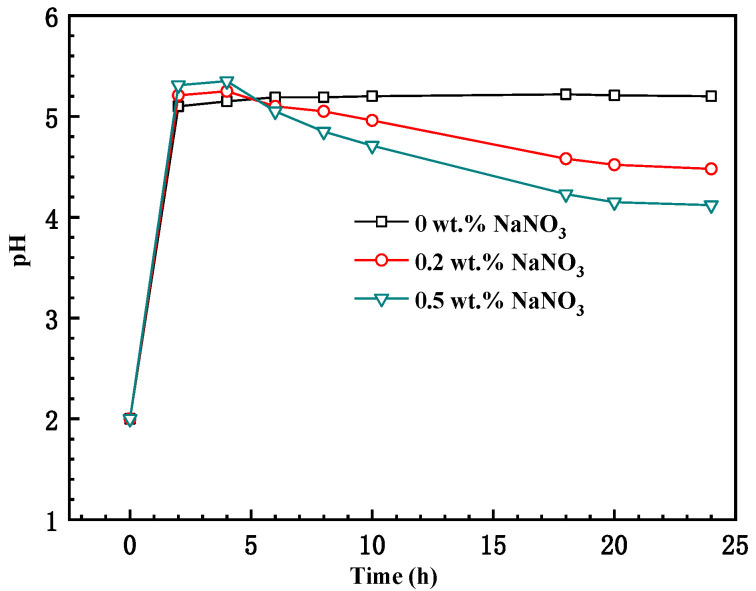
The evolution of the pH inside the crevice in solutions with different concentrations of NaNO_3_.

Figure 7 shows the evolution of the pH inside the crevice in the different test solutions. It can be observed that the pH shows different values inside the crevice in solutions with different concentrations of NaNO_3_. The pH quickly increased from 2 to about 5.1 and gradually stabilized there. The increase in pH could be attributed to hydrogen evolution inside the crevice, which reduces the concentration of H^+^ inside the crevice. The solution inside the crevice become less aggressive than that outside crevice. Thus, the passive film could be stable inside the crevice and contribute to large corrosion resistance in the solution without NaNO_3_, as shown in Figure 4, Figure 5 and Figure 6. In the solution with NaNO_3_, the pH inside the crevice increased in the first 4 h of immersion and then gradually decreased. The pH inside the crevice in the solution with NaNO_3_ was lower than that without NaNO_3_ after corrosion for 24 h. It could be attributed to initiation of crevice corrosion in the solutions with NaNO_3_.

### 3.5. Corrosion Morphology Analysis

**Figure 8:**
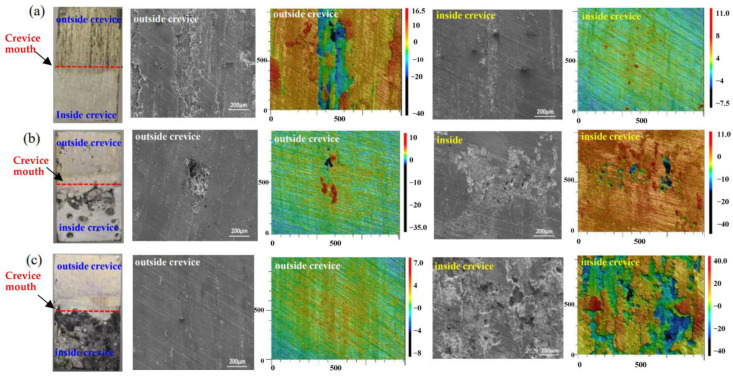
The macro morphology, micro morphology, and 3D depth profiles of specimens inside and outside the crevice in solutions with different concentrations of NaNO_3_ after corrosion for 24 h. (**a**) 0 wt.% NaNO_3_, (**b**) 0.2 wt.% NaNO_3_, (**c**) 0.5 wt.% NaNO_3_.

Figure 8 shows the optical and SEM images of 7075-T651 in solutions with various concentrations of NaNO_3_ after crevice corrosion for 24 h. In the solution without NaNO_3_, the 7075-T651 inside the crevice exhibits slight corrosion, while the 7075-T651 outside the crevice exhibits severe corrosion. This means no active crevice corrosion of 7075-T651 occurred in the solution without NaNO_3_ after 24 h of immersion. In the solution with 0.2 wt.% NaNO_3_, pitting corrosion is detected on the inner and outer specimen surfaces, as shown in Figure 8b. The alloy around the pitting corrosion was hardly attacked on the specimen surface outside the crevice. However, the aluminium alloy around the pitting corrosion gradually began to corrode. Many pitting holes with different depths can be observed on the specimen surface inside the crevice. This could be attributed to the deterioration of the crevice environment, which decreased the stability of the passive film inside the crevice. The specimen outside the crevice maintained its metallic lustre and hardly suffered from corrosion in the solution with 0.5 wt.% NaNO_3_. However, the specimen inside the crevice was covered with a layer of black corrosion product, and severe localized corrosion was observed from the optical and SEM images, as shown in Figure 8c. This means severe crevice corrosion has been initiated in the solution with 0.5 wt.% NaNO_3_ after 24 h of immersion.

### 3.6. Cross-Sectional Morphology

**Figure 9:**
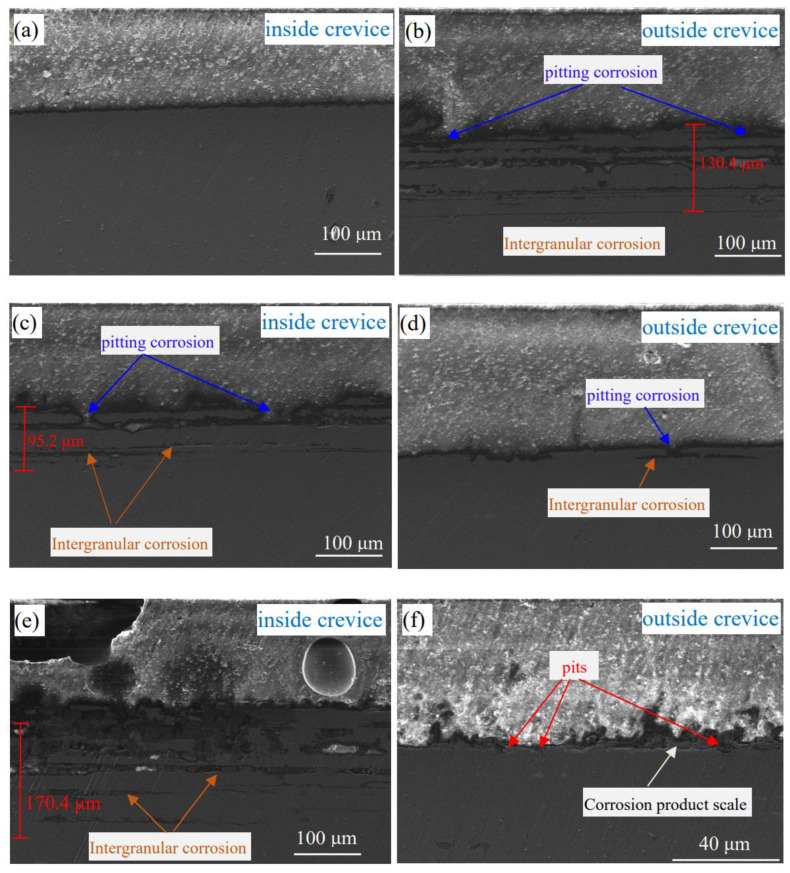
The cross-section of specimens inside and outside crevice in solution with different concentrations of NaNO_3_ after corrosion for 24 h, (**a**,**b**) 0 wt.% NaNO_3_, (**c**,**d**) 0.2 wt.% NaNO_3_, (**e**,**f**) 0.5 wt.% NaNO_3_.

Figure 9 shows SEM images of the cross-sectional morphology of inner and outer aluminium alloys in solutions with various concentrations of NaNO_3_ after corrosion for 24 h. In the solution without NaNO_3_, no apparent corrosion was detected on the aluminium alloy inside the crevice, while serious subsurface intergranular corrosion was detected on the aluminium alloy outside the crevice. The maximum depth of corroded grain boundaries attained was 130.4 μm. In the solution with 0.2 wt.% NaNO_3_, subsurface intergranular corrosion was observed on the specimens inside and outside the crevice. However, the intergranular corrosion of the specimen inside the crevice propagated deeper and wider than that of the specimen outside the crevice, indicating the occurrence of more severe corrosion inside the crevice. Furthermore, the pitting corrosion of the grain boundaries can be observed on both inner and outer aluminium alloys. This means the pitting corrosion can induce subsurface intergranular corrosion. In the solution with 0.5 wt.% NaNO_3_, the aluminium alloy inside the crevice suffered from severe subsurface intergranular corrosion, and the maximum depth of corroded grain boundaries attained was 170.4 μm. However, no obvious pitting and intergranular corrosion was detected on the surface of the aluminium alloy outside the crevice. At the same time, a layer of density corrosion product scale was also observed. Furthermore, it can be seen from Figure 9a–f the corrosion of aluminium alloy inside the crevice is gradually enhanced, and the corrosion of aluminium alloy outside the crevice is progressively inhibited with an increase in NaNO_3_ concentration. This is consistent with the electrochemical measurements. The inhibition effect on specimens outside the crevice could be ascribed to the formation of a protective oxide film with an increased concentration of NaNO_3_. The promoted corrosion of the specimen inside the crevice by NaNO_3_ could be related to the change in chemical species inside the crevice due to the narrow crevice openings limiting diffusion processes; however, the basic reason is unclear.

### 3.7. The Evolution of Localized Corrosion Inside the Crevice

**Figure 10:**
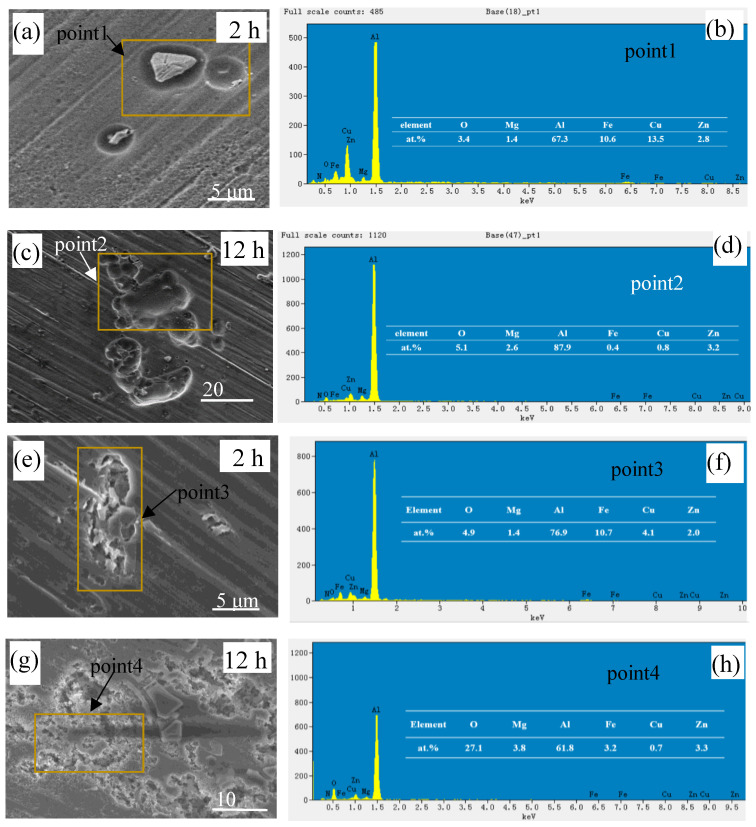
SEM and EDS of localized corrosion area inside the crevice in solutions without and with 0.5 wt.% NaNO_3_ after corrosion fir 2 h and 12 h. (**a**–**d**) 0 wt.% NaNO_3_, (**e**–**h**) 0.5 wt.% NaNO_3_.

The evolution of localized corrosion inside the crevice was analysed using SEM and EDS to explore the effect of NaNO_3_ on the corrosion behaviour of 7075-T651 in solutions without and with 0.5 wt.% NaNO_3_, as shown in Figure 10. It can be seen that elemental Fe and Cu were detected at the localized corroded area after 2 h of immersion in the solution without and with 0.5 wt.% NaNO_3_. This indicates that in the early stage, the localized corrosion inside the crevice is initiated by the intermetallic particles containing elemental Fe and Cu in the solution. However, the morphology of the localized corroded area in the solution without NaNO_3_ was different from that in the solution with 0.5 wt.% NaNO_3_. In the solution without NaNO_3_, the shallow pits were formed at the periphery of intermetallic particles, indicating that the aluminium alloy around intermetallic particles was dissolved simultaneously, perpendicular and parallel to the surface, after 2 h of immersion, as shown in Figure 10a. At an increased immersion time of 12 h, the intermetallic particles gradually detached from the aluminium alloy substrate. Some shallow pits could be observed in the solution without NaNO_3_, as shown in Figure 10c,d. Furthermore, the bottom of these shallow pits maintained a smooth surface, indicating no further corrosion. This means that the corrosion was possibly inhibited when the intermetallic particles detached from the aluminium alloy. In the solution with 0.5 wt.% NaNO_3_, the localized attacked area showed an irregular shape, and deep corrosion grooves or pits could be observed after 2 h of immersion. Furthermore, it could be observed that the aluminium alloy at the interface between the aluminium alloy and the localized corrosion area did not suffer from corrosion, as shown in Figure 10e. This means that the aluminium alloy around intermetallic particles tends to be corroded along the deep direction rather than the surface direction in the solution with 0.5 wt.% NaNO_3_. At an increased immersion time of 12 h, serious localized corrosion could be observed in the solution with 0.5 wt.% NaNO_3_, as shown in Figure 10g. Furthermore, elemental Cu and Fe could still be detected in the localized corrosion area. This indicates that the intermetallic particles still adhered to the aluminium alloy surface after 12 h immersion.

## 4. Discussion

The crevice corrosion mechanism of 7075-T651 in solution with NaNO_3_.

According to Figure 10a,b, the localized dissolution of the aluminium alloy inside the crevice was enhanced due to the micro-galvanic corrosion induced by intermetallic particles. This resulted in a negative shift of OCPs of the specimens inside the crevice within 4 h of immersion (Figure 3a) in the acidic NaCl solution without NaNO_3_. However, the intermetallic particles gradually detached from the specimens’ surface inside the crevice at prolonged immersion time, which resulted in the disappearance of the galvanic corrosion effect, as shown in Figure 10c,d. Moreover, it can be seen from Figure 3 and Figure 4 that the corrosion potential of 7075-T651 inside the crevice lies in the passive region of the polarization curve. Thus, an intact oxide film is formed on the specimen surface when the intermetallic particles are detached from the specimen. This inhibits the corrosion of the specimen inside the crevice and results in the positive shift of OCP of the specimen inside the crevice after 4 h of immersion (Figure 3) in an acidic NaCl solution without NaNO_3_.

In the solution with NaNO_3_, NaNO_3_ plays a controversial role in the corrosion of aluminium alloys inside and outside the crevice. The NaNO_3_ assists in the formation of a protective oxide film and reduces the corrosion of the aluminium alloy outside the crevice. At the same time, it causes severe localized corrosion of the aluminium alloy inside the crevice. Beyond all doubt, the change in chemical species inside the crevice is mainly responsible for initiating localized corrosion in solution with NaNO_3_.

Furthermore, it has been reported [19] that NO_3_^−^ could participate in the cathodic reactions and eventually be reduced to NH_3_. This leads to the accumulation of NH_3_ inside the crevice hole in the solution with NaNO_3_. This demonstrates that the NH_3_ is a potential factor in initiating crevice corrosion of 7075-T651. Thus, an experiment was performed to determine the effect of NH_3_ on the crevice corrosion of 7075-T651, and the test setup is shown in Figure 2. In order to minimize the effect of the pH, the pH of all simulated crevice solutions was adjusted to 5.2 as depicted in Figure 7. As a result, two kinds of simulated crevice solutions were prepared, which were the 3.5 wt.% NaCl + 0.5 wt.% NaNO_3_ solution with pH = 5 and the 3.5 wt.% NaCl + 0.25 wt.% NH_4_NO_3_ solution with pH = 5, respectively. The simulated bulk solution was the 3.5 wt.% NaCl + 0.5 wt.% NaNO_3_ solution with pH = 2.

**Figure 11:**
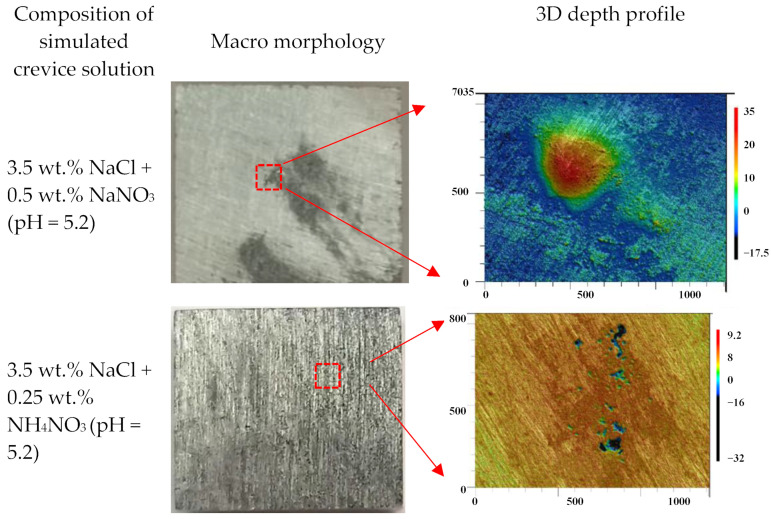
The macro morphology and 3D depth profiles of specimens in simulated crevice solution after corrosion for 24 h.

**Figure 12:**
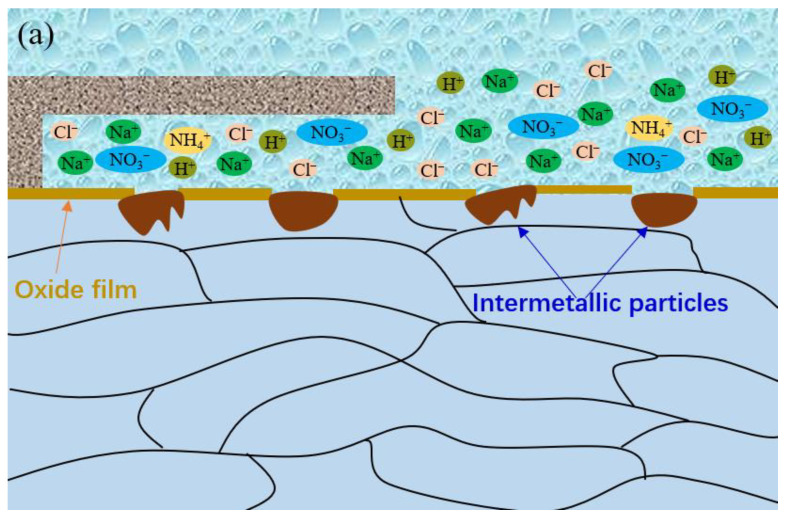

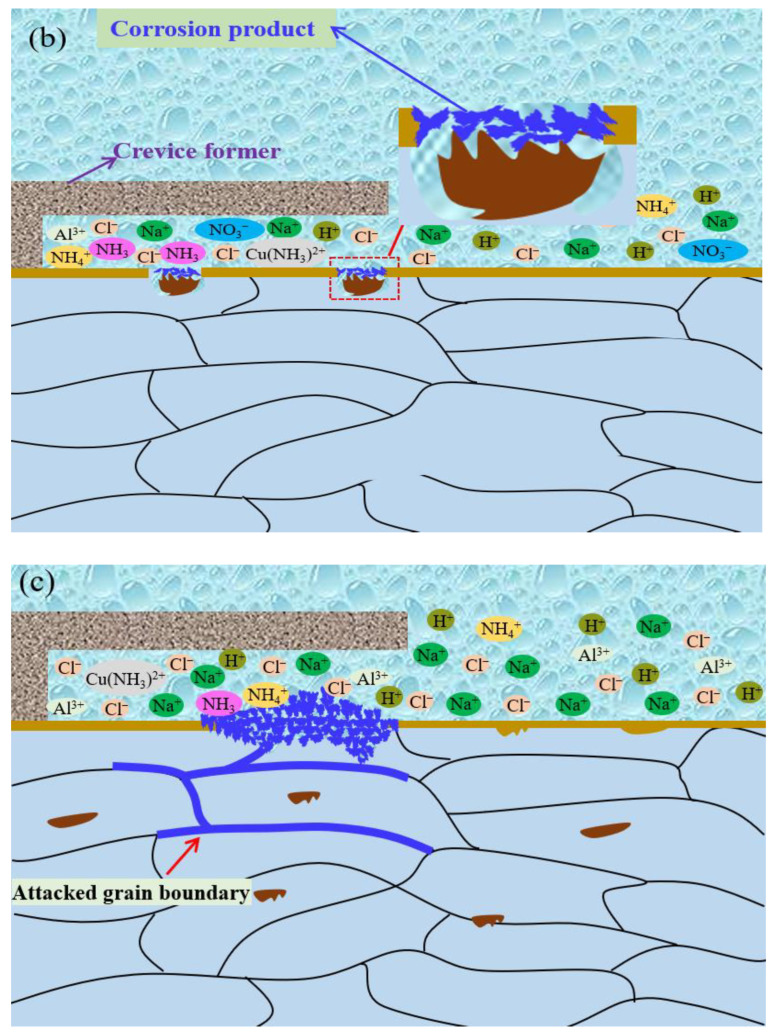
The crevice corrosion mechanism of the 7075-T651 aluminium alloy in acidic NaCl with NaNO_3_, (**a**) the induction period, (**b**) the quick development period, (**c**) the stable development period.

Figure 11 shows the corrosion morphology after 24 h of immersion. It can be seen that only a bulge point could be observed, and no obvious pitting corrosion was detected on the specimen surface in the simulated crevice solution containing 0.5 wt.% NaNO_3_. However, obvious pitting corrosion could be detected on the specimen surface in the simulated crevice solution containing 0.25 wt.% NH_4_NO_3_. This indicates the presence of NH_4_^+^ could promote the nucleation of pitting corrosion of 7075-T651. Thus, it can be deduced that the accumulation of NH_3_ inside the crevice due to the reduction of NO_3_^−^ is mainly responsible for initiating crevice corrosion of 7075-T651. Figure 12 describes the crevice corrosion mechanism of 7075-T651 in an acidic NaCl solution with NaNO_3_. The NO_3_^−^ promotes the formation of an oxide film on the 7075-T651 surface, which inhibits the galvanic corrosion between intermetallic particles and the aluminium alloy, as shown in Figure 12a. This leads to a positive shift of the corrosion potential of the specimen inside the crevice at the early stage, as shown in Figure 3b,c. As the corrosion progresses, NH_3_ gradually accumulates at the crevice hole due to the reduction of NO_3_^−^. It has been reported that NH_3_ and Cu^2+^ form stable complexes Cu(NH3)_2_^2+^ in solution [19,26]. This suggests that the Cu of intermetallic particles is selectively dissolved by accumulated NH_3_ inside the crevice, which contributes to forming irregular corrosion morphology, as shown in Figure 10e. The aluminium alloy at the interface between intermetallic particles and aluminium alloy substrate is initially exposed to a crevice solution followed by micro-galvanic corrosion due to the dissolution of Cu. However, the exposed aluminium alloy could be re-passivated at the early stage. As the corrosion progresses, the process of exposure and re-passivation of the aluminium alloy could be repeated. This generates deep corrosion grooves or pits at the interface between the intermetallic particles and the aluminium alloy substrate, as shown in Figure 10e and Figure 12b. With the increased depth of corrosion grooves or pits, the transportation of chemical species could be limited and result in the accumulation of metal ions inside the grooves or pits. Then, the H^+^ and Cl^−^ inside the grooves or pits are enriched, resulting in a hydrolysis reaction and electric neutrality.

Moreover, it is difficult for NO_3_^−^ to diffuse into the bottom of the deep grooves or pits. Thus, with the deterioration of the environment inside the deep grooves or pits, the localized corrosion is gradually stabilized. The metal ions inside the localized pits gradually migrate to the crevice solution, resulting in an increased concentration of H^+^ and Cl^−^ in the crevice solution near localized corrosion pits. Subsequently, the passive film near the localized pits is preferentially destroyed, as shown in Figure 8 and Figure 12c. Furthermore, according to previous reports [27,28,29], Cu is usually absent at the grain boundary, and this chemical heterogeneity results in high corrosion activity at the grain boundary. Thus, the exposed grain boundary is preferentially corroded, leading to serious subsurface intergranular corrosion, as shown in Figure 9 and Figure 12c.

## 5. Conclusions

1. NO_3_^−^ plays a crucial role in the crevice corrosion of 7075-T651. Crevice corrosion occurs in acidic NaCl solution with NO_3_^−^ and does not occur in acidic NaCl solution without NaNO_3_.
2. The corrosion of the specimen inside the crevice is promoted, and the corrosion of the specimen outside the crevice is inhibited due to increased NO_3_^−^ concentration.
3. The secondary product, NH_3_, induces pitting nucleation inside the crevice and then initiates crevice corrosion due to the reduction of NO_3_^−^.

## Figures and Tables

**Table 1:** The fitted electrochemical parameters for EIS of 7075-T651 inside the crevice and outside the crevice in acidic NaCl solutions with different concentrations of NaNO_3_.

*C_NaNO_3__* (wt.%)		Rs (Ω cm^2^)	CPE_ox_ (Ω^−1^ cm^−2^ s^−n1^)	n1	R_ox_ (Ω cm^2^)	CPE_ct_ (Ω^−1^ cm^−2^ s^−n2^)	n2	R_ct_ (Ω cm^2^)	L (H/cm^2^)	R_L_ (Ω cm^2^)	R_p_ (Ω cm^2^)
0	Out	7.51	2.84 × 10^−5^	1	32.58	3.44 × 10^−5^	0.84	436.3	637.8	1570	374
	In	1.77	1.75 × 10^−5^	0.89	2889	0.0189	1	3226			6115
0.2	Out	6.88	4.32 × 10^−5^	0.96	964	5.80 × 10^−5^	0.88	255	445	1025	1168
	In	2.13	2.81 × 10^−5^	0.85	926	8.05 × 10^−5^	0.78	11.43	264.5	858	937.07
0.5	Out	7.62	9.51 × 10^−5^	0.84	990.3	0.039	1	706.5			1696.8
	In	2.35	4.95 × 10^−5^	0.75	677.2	2.02 × 10^−5^	0.86	36.61	233.2	337.6	710.00

## Data Availability

The data that support the findings of this study are available on request from the corresponding author.
